# Tight Junction Disruption Induced by Type 3 Secretion System Effectors Injected by Enteropathogenic and Enterohemorrhagic *Escherichia coli*

**DOI:** 10.3389/fcimb.2016.00087

**Published:** 2016-08-24

**Authors:** Paul Ugalde-Silva, Octavio Gonzalez-Lugo, Fernando Navarro-Garcia

**Affiliations:** Department of Cell Biology, Centro de Investigación y de Estudios Avanzados del Instituto Politécnico NacionalMéxico City, Mexico

**Keywords:** cell junction, occludin, claudin, ZO-1, Enteropathogenic *E. coli*, Enterohemorrhagic *E. coli*, T3SS effectors, tight junction

## Abstract

The intestinal epithelium consists of a single cell layer, which is a critical selectively permeable barrier to both absorb nutrients and avoid the entry of potentially harmful entities, including microorganisms. Epithelial cells are held together by the apical junctional complexes, consisting of adherens junctions, and tight junctions (TJs), and by underlying desmosomes. TJs lay in the apical domain of epithelial cells and are mainly composed by transmembrane proteins such as occludin, claudins, JAMs, and tricellulin, that are associated with the cytoplasmic plaque formed by proteins from the MAGUK family, such as ZO-1/2/3, connecting TJ to the actin cytoskeleton, and cingulin and paracingulin connecting TJ to the microtubule network. Extracellular bacteria such as EPEC and EHEC living in the intestinal lumen inject effectors proteins directly from the bacterial cytoplasm to the host cell cytoplasm, where they play a relevant role in the manipulation of the eukaryotic cell functions by modifying or blocking cell signaling pathways. TJ integrity depends on various cell functions such as actin cytoskeleton, microtubule network for vesicular trafficking, membrane integrity, inflammation, and cell survival. EPEC and EHEC effectors target most of these functions. Effectors encoded inside or outside of locus of enterocyte effacement (LEE) disrupt the TJ strands. EPEC and EHEC exploit the TJ dynamics to open this structure, for causing diarrhea. EPEC and EHEC secrete effectors that mimic host proteins to manipulate the signaling pathways, including those related to TJ dynamics. In this review, we focus on the known mechanisms exploited by EPEC and EHEC effectors for causing TJ disruption.

## Introduction

The intestinal epithelium is the largest mucosal surfaces of the body, covering about 400 m^2^ of surface area with a single layer of cells organized into crypts and villi. This surface is continually renewed by pluripotent intestinal epithelial stem cells that reside in the base of crypts (van der Flier and Clevers, 2009). The intestinal epithelium constitutes the most important barrier against the external environment, represented by the intestinal lumen (Farhadi et al., 2003). The epithelium acts as a selectively permeable barrier, permitting the absorption of nutrients, electrolytes, and water contained in the intestinal lumen, while maintaining an effective defense against intraluminal toxins, antigens, and enteric flora. The intestinal epithelium contains five distinct types of cells: stem cells with interposed Paneth cells producing antimicrobial peptides, absorptive enterocytes, goblet cells secreting mucus, and enteroendocrine cells producing hormone (Kraehenbuhl et al., 1997). The intestinal epithelium mediates selective permeability through two major pathways, the transcellular and paracellular route (Tsukita et al., 2001). Transcellular permeability is generally associated with solute transport through the epithelial cells and predominantly regulated by selective transporters for amino acids, electrolytes, short-chain fatty acids, and sugars (Ferraris and Diamond, 1997; Kunzelmann and Mall, 2002; Broer, 2008). Paracellular permeability is associated with the transport in the space between epithelial cells, and it is regulated by intercellular complexes localized at the apico-lateral membrane junction and along the lateral membrane (Van Itallie and Anderson, 2006).

**Figure 1:**
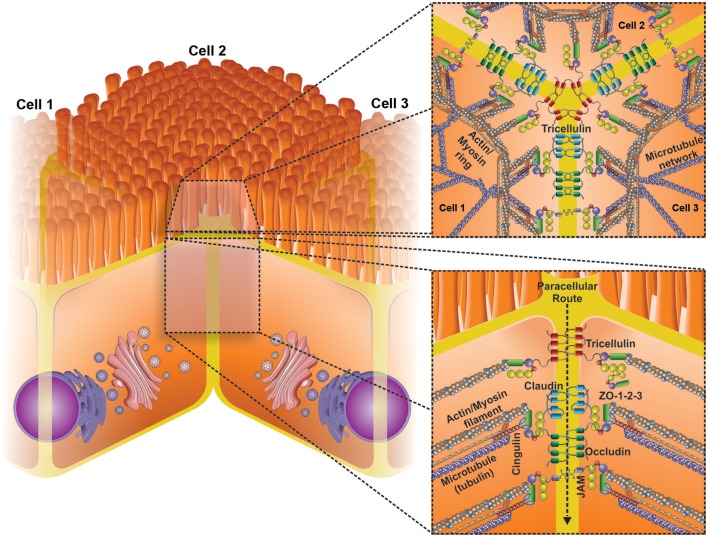
**Schematic representation of the basic structural transmembrane and peripheral membrane components of tight junctions (TJs)**. TJs are the most apical intercellular junctions in epithelial cells (lower insert). TJs consist of a complex interaction of three core protein components: (i) transmembrane proteins (occludin, claudins, junctional adhesion molecules [JAM] and tricellulin) (ii) Accessory proteins (Zonulae occludins 1–3), and (iii) cytoskeletal proteins (actin etc.). Changes in the expression and location of these proteins can lead to significant changes in the epithelial barrier function. Upper insert shows a transversal view of three cells bound by TJs, highligting the role of tricellulin junction.

Epithelial cells are held together by the apical junctional complexes, consisting of adherens junctions (AJs) and tight junctions (TJs), and by underlying desmosomes (Suzuki, 2013). The AJs, along with desmosomes, provide strong adhesive bonds between the epithelial cells, and also help intercellular communication, but do not determine paracellular permeability (Nekrasova and Green, 2013). The TJs circumscribe the apical ends of the lateral membranes of epithelial cells and determine the selective paracellular permeability to solutes (Van Itallie and Anderson, 2006). Thereby, the TJs provide both a barrier to harmful molecules and a pore for the permeation of ions, solutes, and water as appropriate. TJs are comprised of four transmembrane proteins, claudins (Furuse et al., 1998), occludin (Furuse et al., 1993), junctional adhesion molecules (JAMs) (Martin-Padura et al., 1998), and tricellulin (Ikenouchi et al., 2005), and some cytosolic scaffold proteins, such as zonulae occludens (ZO), and cingulin (Lee, 2015). TJ proteins interact with the actin cytoskeleton for maintaining TJ structure (Figure 1) and this interaction permits the cytoskeletal regulation of TJ barrier integrity (González-Mariscal et al., 2003). At the same time, the circumferential contraction and tension in the actin is regulated by myosin light chain (MLC) activation (Turner et al., 1997). MLC phosphorylation induced by kinases such as MLC kinase (MLCK) and Rho-associated kinase causes the contraction of the actin, resulting in the opening of the paracellular pathway (Walsh et al., 2001). Intestinal permeability is a dynamic process and its regulation is determined by the interaction among several participants, such as barrier constituents, immune cells, microbiota, and also external factors, including the diet. Alterations of mucosal barrier function are increasingly linked to a broad spectrum of pathologies, such as nonalcoholic fatty liver disease (Miele et al., 2009), type 1 diabetes (Bosi et al., 2006) and chronic intestinal inflammation (Pastorelli et al., 2013).

Thus, through all these protein complexes, the TJs are found in the most apical part of the epithelial cells restricting, among other factors, the entry of most of the microorganisms living or colonizing the intestinal mucosa. However, diverse pathogens have generated a number of strategies to disrupt these protein structures and spread to various tissues. Different pathogens produce virulence factors associated to TJ disruption. For instance, viruses as the Hepatitis C use occludin in the initial steps of their internalization (Ploss et al., 2009), or claudin-1 in the last steps of virus internalization (Evans et al., 2007). Fungi as *Aspergillus* and *Penicillium* produce ochratoxin A, which disrupts the epithelium permeability by removing specific isoforms of claudin from the TJs (McLaughlin et al., 2004). Parasites as *Entamoeba histolytica* produce a cysteine protease (EhCP-A5), which induces a proinflammatory response that is related to an increase in the TJ permeability (Kissoon-Singh et al., 2013). Manipulation of TJs is not exclusive of viruses, fungi or parasites, pathogenic bacteria also produce virulence factors for targeting the TJs. For example, *Helicobacter pylori* injects, through the type 4 secretion system, the CagA protein into the cytoplasm of gastric epithelial cells. CagA associates with ZO-1 and JAM causing assembly of TJ components at the sites of attached bacteria, altering the composition and function of the apical junction complex (Amieva et al., 2003). Other bacterial pathogens use similar strategies for translocating proteins, called effector proteins, to the cytoplasm of the host cells to disrupt the TJ integrity. Enteropathogenic and enterohemorrhagic *Escherichia coli* (EPEC and EHEC) are members of a family of bacterial pathogens with the capability of causing a histopathological lesion called attaching and effacing lesion (A/E lesion). Bacterial pathogens causing this lesion are collectively named as A/E pathogens and also include *Citrobacter rodentium, Hafnia alvei* and EPEC affecting animals such as rabbit (REPEC). A/E lesions are characterized by effacement of the brush border microvilli, intimate bacterial adherence to the enterocyte apical plasma membrane, and the accumulation of polymerized actin beneath the attached bacteria (Knutton et al., 1987). In order to manipulate the actin cytoskeleton, EPEC, and EHEC inject, by a type 3 secretion system (T3SS), effector proteins, which are able to hijack the host cell machinery interfering with host cell pathways and with a number of actin binding proteins. The actin cytoskeleton is a dynamic structure necessary for cell and tissue organization (Navarro-Garcia et al., 2013), including the maintenance of epithelial barriers.

For generating the A/E lesion or pedestal-like structure, EPEC and EHEC manipulate transduction signaling pathways of the host cells to produce this lesion. EPEC and EHEC use various proteins that are encoded in a chromosomal pathogenicity island (PAI) known as the locus of enterocyte effacement (LEE). This PAI is shared by the other A/E pathogens and encodes factors needed for the pathogenesis of EPEC/EHEC, including the diarrhea production (McDaniel and Kaper, 1997). Inside LEE PAI are genes encoding for proteins of the T3SS, including translocator proteins such as EspA, EspB, and EspD (Hartland et al., 2000), which are used by EPEC/EHEC to inject effector proteins inside the host cell. LEE also encodes for effector proteins such as Tir, EspF, EspG, EspZ, EspH, and Map (Wong et al., 2011), gene regulators such as Ler (LEE-encoded regulator) (Elliott et al., 2000), chaperone proteins such as CesT for Tir, (Abe et al., 1999), CesD for EspB and EspD (Wainwright and Kaper, 1998), and CesF for EspF (Elliott et al., 2002). Additional to LEE effector proteins, exist other effector proteins that are encoded outside of LEE, known as non-LEE encoded effectors, such as NleA/EspI, NleB, NleC, NleD, NleE, EspJ, NleH, EspG, EspM, and Cif (Dean and Kenny, 2009; Vossenkamper et al., 2011; Salvador et al., 2014).

Most of the subversions by these effectors are related to the cell cytoskeleton modification, TJs disruption, cell death, and inflammation. Various effector proteins mimic epithelial cell proteins through different cellular pathways. In this review, we will focus on the molecular strategies used by EPEC and EHEC to disrupt the TJs.

## Disassembly of TJs by EPEC and EHEC

Classically, TJs integrity is evaluated by measuring the transepithelial electrical resistance (TEER). The electrical impedance allows measuring the continuous current passing through the cells on both transcellular and paracelllular pathways. The transcellular resistance is due to the apical and basolateral plasma membrane, while the paracellular resistance results of cell-substrate and cell-cell contacts. As mentioned above, specific TJs proteins principally influence the epithelial resistance. Thereby, the TEER values reflects the electrophysiological properties due to the physical structures and properties of filter-grown epithelial cultures (Chen et al., 2015). Thus, TEER measurement has been one of the main tools for determining the permeability of TJs or membrane perturbation by disruptors of intestinal and kidney epithelial cells lines (Madgula et al., 2007).

It has been described that EPEC/EHEC alter the TJ permeability and thereby the TEER. EPEC infection using T84 monolayers showed that bacteria are able to disrupt the TEER due to the TJ disruption by assessing the mannitol flux (Spitz et al., 1995). These defects in the epithelial barrier induced by EPEC infection cause an increase of the monolayer permeability and atypical distribution of ZO-1 (Philpott et al., 1996). Subsequently to this atypical distribution of ZO-1 induced by EPEC, other TJ proteins as occludin and claudin have been found to be redistributed. In fact, EPEC infection induces occludin dephosphorylation and its relocalization from TJs to intracellular compartments of T84 cells (Simonovic et al., 2000). Similarly, claudin distribution is affected by EPEC infection in T84 cells (Muza-Moons et al., 2004) and by using a murine model *in vivo* infected with EPEC (Zhang et al., 2012).

Like EPEC, EHEC generates a TEER decrease, and this decrease is independent of the Stx action. Indeed, T84 monolayers treated with purified Stx do not show a TEER decrease; however, damage to barrier function is used as a form of the toxin translocation (Philpott et al., 1997). Redistribution of TJ proteins is also found during the infection of EHEC. Contrary to the distribution of ZO-1 and TJ disruption, there is no disruption of the AJ protein E-cadherin during the infection by EHEC (Philpott et al., 1998). Interestingly, an acute model of infection by EHEC showed a rapid disruption of TJs, while the chronic infection by EHEC produced a change in the expression of claudins, favoring a phenotype of high permeability, which was independent of Stx1 (Roxas et al., 2010).

**Figure 2:**
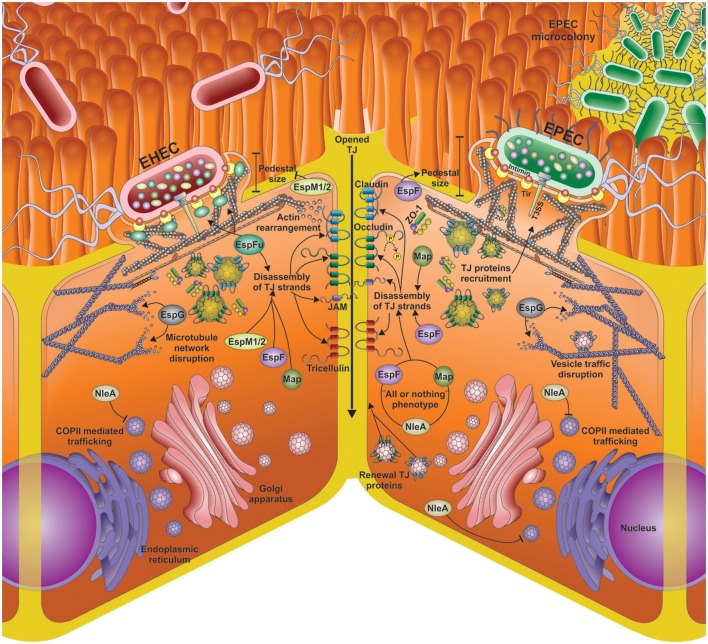
**Different mechanisms for tight junction (TJs) opening by EPEC and EHEC effector proteins**. Disassembly of TJs by the main LEE and non-LEE effectors injected by the EHEC and EPEC T3SS into the epithelial cells. The main mechanisms for TJ disassembly include actin rearrangement, occludin de-phosphorylation, microtubule network disruption; COPII mediated trafficking inhibition, vesicle traffic disruption, and inhibition of the TJ proteins renewal. Besides the shared effectors (EspF, EspG, Map, NleA) by EHEC and EPEC, EHEC infection (left side) also includes EspFu and EspM1/2.

The ability of EPEC/EHEC for decreasing TEER and causing altered distribution of TJ proteins has been attributed to diverse proteins encoded in the LEE PAI. The insertion of the LEE PAI in a non-pathogenic *E. coli* K12 confers the capability for producing changes in the localization of occludin. Moreover, a mutant strain in structural genes of the T3SS is unable to cause the altered localization of occludin (Simonovic et al., 2000). These data suggest that the genes encoded in LEE, as well as a functional T3SS are required to produce changes in TJs integrity. Additionally, some effectors encoded in LEE such as EspF, Map, and EspG, as well as non-LEE effectors, such as NleA and EspM, have been involved in the altered distribution of TJ proteins (Figure 2), and thereby in the production of diarrhea due to the infection by EPEC and EHEC.

## Effectors encoded in LEE

### EspF

*E. coli* secreted protein F (EspF) was identified based on the proline-rich repeats (PRR) in its amino acid sequence, very similar to eukaryotic motifs. Each of these PRRs comprises a putative motif of *src* homology 3 (SH3) ligand binding domains, which have been reported that mediate protein interaction and are found in a variety of eukaryotic proteins that participate in various signaling networks (McNamara and Donnenberg, 1998). On the other hand, EHEC produces various effector proteins with similarity to EPEC EspF including EHEC EspF (87% of similarity), EspFu/TccP (24% of similarity), and M-EspF (28% of similarity). Another important difference among these proteins is the number of PRR; EHEC EspF contains four, while EspFu and M-EspF contain seven and five, respectively (Viswanathan et al., 2004). It has been found that these PRR motifs specifically interact with SH3 domains of the eukaryotic protein SNX9 (Sorting Nexin 9), inducing tubules formation in the host cell membrane (Alto et al., 2007). Although the function of these tubules has not been discovered, Weflen et al. proposed that EPEC EspF uses SNX9 to promote the invasion of neighboring epithelial cells (Weflen et al., 2010). Within the PRR sequence of EPEC EspF there is also a putative domain like the binding domain to Cdc42/Rac (CRIB) of N-WASP. EspF PRRs stimulate actin nucleation mediated by the N-WASP/Arp2/3, possibly by binding to CRIB autoregulation domain of N-WASP (Alto et al., 2007).

In recent years EPEC and EHEC EspF have been classified as multifunctional bacterial effectors that manipulate diverse cellular processes such as antiphagocytic processes, elimination of intestinal microvilli, remodeling of the host cell membrane, modulation of the cytoskeleton dynamics, destruction of the nucleolus, elimination of intermediate filamentous, cellular invasion, mitochondrial dysfunction, cell death by apoptosis, inhibition of important epithelial transporters, as well as disruption of the epithelial barrier (Holmes et al., 2010).

In 2001, MacNamara et al. reported the first finding relating EspF with disruption of epithelial barrier; an *espF* mutant was deficient for disrupting TEER of T84 monolayers. This *espF* mutant was also unable to induce increase in epithelial flux of mannitol, a marker of paracellular permeability, and was unable to distribute occludin from the TJs (McNamara et al., 2001). On the other hand, EHEC EspF appears to play a more moderate role in the transepithelial barrier, since neither a mutation on *espF* nor *cesF* (gene for the EspF chaperone) in EHEC prevented the loss of TEER induced by EHEC. However, EHEC EspF and EspFu complemented to an *espF* mutant in EPEC (EPECΔ*espF*) for restoring the ability to disrupt the TEER and occludin redistribution. Additionally, it has been noticed that EspFu increases the TJ disruption, since when EHEC is transformed with a high expression plasmid containing *espFu/tccp*, the high expression of this protein increases the TJ disruption in comparison with the EHEC wild type strain (Viswanathan et al., 2004).

During EPEC infection on T84 monolayers, TJ perturbation can result from disruption of protein-protein interactions. Although, the protein concentration is kept constant, EPEC produces a disorganized distribution of claudin 1, occludin and ZO-1, which leads to progressive loss of TEER. This loss in TEER is correlated with aberrant strands in the lateral membrane (Muza-Moons et al., 2004). These data have been consistent using an *in vivo* mouse model. EPEC wild type caused occludin redistribution and considerably diminished the intestinal barrier functions of the ileum and colonic mucosa (Shifflett et al., 2005). Thus, EPEC opens the TJ strands in an EspF-dependent way, a process that is extremely related to diarrhea production.

Diverse stimulus, including calcium reduction, TNF-α, IFN-γ, and epidermal growth factor (EGF), rapidly modify the structure of the TJ strands. The dynamic behavior of TJs includes the movement of TJ strands and of individual TJ proteins, which can migrate within the TJ and in and out of these structures. Some TJ proteins are continually endocytosed and recycled back to the plasma membrane. Regulation of the endocytic trafficking of TJ proteins may provide a rapid way for remodeling of TJs (Chalmers and Whitley, 2012). In this context, during an infection, EPEC is able to manipulate vesicular trafficking dynamics of TJ proteins not only for destabilization of TJ strands but also for growing of actin-rich pedestals. A clear example is ZO-1, which is generally accumulated in the intracellular region of the TJ but not in other actin-rich structures such as stress fibers or filopodia. However, it was reported that during EPEC infection, ZO-1 is recruited to the actin-rich pedestals, and this incorporation was dependent of proline-rich region of ZO-1 (Hanajima-Ozawa et al., 2007). Additionally, in a model using RK13 cells from rabbit kidney, other TJ proteins have been found in the actin-rich pedestals induced by REPEC. REPEC EspF causes disruption of TJ strands and the recruitment of the transmembrane proteins claudin and occludin and the proteins from the cytoplasmic plaque ZO-1 and ZO-2 to the actin-rich pedestals. Interestingly, *espF* mutant in REPEC does not cause disruption of the TJ strands and as a consequence, the cells produce smaller pedestals than those induced by the EPEC wild type. Although the exact mechanism of EspF-mediated TJ protein redistribution is yet to be defined, *in silico* analyses have revealed that EspF contains putative SH3 and PDZ domain binding motifs, which may have a crucial role in the interaction with some promoter factors of actin nucleation as well as with proteins of the TJ containing PDZ domains. Indeed, REPEC EspF interacts with profilin, N-WASP, as well as with ZO-1 and ZO-2 and with actin. It has been suggested that the interaction of REPEC EspF with these nucleation factors may be related to an imbalance in actin polymerization-depolymerization cycles and in this way, being related to endocytosis of TJ proteins (Peralta-Ramirez et al., 2008).

In a similar way, *in silico* analyses and yeast double hybrid assays, Blasche et al. found a group of proteins associated to actin that interact with EHEC EspF, such as Arp2, profilin, N-WASP, and ZO-1/ZO-2. Additionally, various proteins containing BAR-like domains interact with EHEC EspF such as SNX9 and SNX33 (Blasche et al., 2014). A relationship between proteins with BAR-like domains and the pedestal maturation induced by EHEC has been recently found. TOCA1, FBP17, and CIP4 are BAR domain containing proteins that regulate membrane dynamics through the ability for causing membrane tubulation. Campellone et al. found that TOCA1 positively contributes in the pedestal biogenesis mediated by EspFu, while FBP17 (probably CIP4) plays an inhibitory role in the pedestal assembly. The interaction of EspFu with SH3 domain of TOCA1 is implicated in an increased actin polymerization by N-WASP (Campellone et al., 2012). Additionally, it has been demonstrated that some proteins regulating the membrane dynamics, mainly TOCA1, also regulate TJ assembly. Van Itallie et al. found that PDZ domain of TOCA1 interacts with ZO-1, leading to N-WASP and its actin regulation machinery directly to the TJs. Besides, the TOCA1 knockout results in an increase of the paracellular flux and suppresses the membrane junction dynamics, which are believed to be required for keeping the seals in the cell-cell contacts (Van Itallie et al., 2015).

### Map

The mitochondrial associated protein (Map) is an effector dependent on T3SS, which was detected in supernatants from EPEC wild type strain, but not in supernatants from a strain mutant in *orf19* gene or in a strain lacking the T3SS. Once inside the mitochondria, Map causes disruption of the mitochondrial membrane potential in HeLa cells. Map also interferes with ATP generation, inhibiting the oxidative phosphorylation that is mediated by the enzyme succinate dehydrogenase (SDH) in a murine model and *C. rodentium*. Thus, Map activity contributes in the intestinal mucosa damage through destabilizing the mitochondrial structure, as well as the respiratory chain of infected epithelial cells (Ma et al., 2006).

Additionally, Map transiently directs the formation of filopodia in the EPEC adherence site on the HeLa cell membrane, a process that is independent of the mitochondrial localization. Also, Map contains a classical motif of PDZ1 binding domain, with the consensus sequence VQDTRL, which is implicated in microvilli remodeling in Caco-2 cells infected with EPEC. This motif is related to the interaction of Map with the protein Ebp50/NHERF1 (ezrin/radixin/moesin (ERM)-binding phosphoprotein 50/Na^+^/H^+^ exchanger regulatory factor 1). Ebp50 is one of the surface microvilli components that directs membrane receptors to the apical membrane, specifically, it directs Map to the plasma membrane by using the motif VQDTRJ, a PDZ ligand of Map. Once in the membrane, Map uses a novel motif of bacterial origin with the consensus sequence WxxxE to mimic GTPases that activates small G protein Cdc42, which in turn activates filopodia formation mediated by EPEC (Kenny et al., 2002; Alto et al., 2006). These initiating events between Ebp50 and Map trigger an actin-based positive feedback loop, leading to initial Cdc42 polarization and a subsequent burst of actin polymerization at the site of bacterial infection (Orchard et al., 2012). As demonstrated by Berger et al. Map has the same function in EPEC and EHEC (Berger et al., 2009).

Map also has the ability to induce other effects related to diarrhea production. EPEC mutated in *map* gene was strongly deficient for decreasing TEER, in comparison with the EPEC wild type that rapidly decreased the TEER. This effect of Map on the TEER is independent of Map localization in the mitochondria (Dean and Kenny, 2004). Occludin forms a continuous perimeter band in the apical region, and this distribution is kept in cells infected with a EPEC strains with a mutation in *map* gene, implicating Map in the epithelial barrier dysfunction mediated by EPEC and giving to Map an important role in the diarrhea produced by EPEC (Dean and Kenny, 2004).

### EspG

The *espG* gene encodes for a protein that is secreted and translocated by the T3SS. *espG* is highly conserved in LEE from EPEC (E2348/69) and EHEC O157:H7 with an identity of 98%. EspG protein also has a significant homology (21% identity and 40% similarity) with VirA, a protein secreted by the T3SS of *Shigella flexneri*. Additionally, there is an EspG homolog, named EspG2, which is encoded far away from LEE (Elliott et al., 2001).

The important role of EspG of EHEC as a regulator of endomembrane trafficking has been reported. For carrying out this process, EspG uses as substrates the eukaryotic proteins ARF (ADP-ribosylation factor), a GTPase related to the organization of the vesicle transport, as well as uses as substrate PAK proteins (p21-activated kinases), which are implicated in the signal transduction mediated by the GTPases Cdc42 and Rac1 that establish intracellular polarity (Germane and Spiller, 2011; Selyunin et al., 2011). EspG targets ARF in the active conformation, ARF-GTP. EspG-ARF interaction blocks the access of GAPs (GTPase activating proteins), preventing the hydrolysis of ARF-GTP in the γ-phosphate of GTP and perturbing the normal cycling of guanines (Selyunin et al., 2011). Additionally, EspG-ARF-GTP forms a ternary complex with Rab1, severely interrupting the host secretory pathway. EspG harbors a TBC-like dual-finger motif and exhibits potent RabGAP activities (Dong et al., 2012). The interaction of PAK2 with EspG is strictly dependent on the EspG-ARF1-GTP complex and ARF tethering to the membrane. Once these three factors are bound, PAK2 activity increases 7.6 times. EspG-PAK interaction blocks the binding to PAK substrates, by blocking the catalytic site, thereby stabilizing the homodimer structure (Selyunin et al., 2011). The interaction of EspG with Rac/Cdc42(p21) binding domain of PAK1, mimics a small GTPase, implying that the primary role of EspG during pathogenesis is to promote actin remodeling. Rather than recruiting a small GTPase, EPEC circumvent the standard PAK activation mechanism (Germane and Spiller, 2011). EspG, by mimicking the function of diverse small GTPases related to the actin cytoskeleton, is able to alter the balance of the formation of diverse actin structures (Hardwidge et al., 2005).

Interestingly, EspG exploits GEF-H1 (a nucleotide exchanger factor) for inducing stress fibers formation during EPEC infection. EspG is associated to actin, resulting in the disruption of the microtubules network. GEF-H1 changes to its active form as a result of its dissociation of the microtubules network. Active GEF-H1 promotes the GTP binding to RhoA, resulting in RhoA activation. Activation of ROCK, that is localized downstream of the RhoA signaling pathway, induces the actin stress fibers assembly (Matsuzawa et al., 2004).

Although, the exact mechanism by which EspG induces microtubule disassembly is unknown yet, it has been reported that EspG directly interacts with tubulin (Hardwidge et al., 2005). EspG-tubulin interaction was also detected in epithelial cells and correlated with the induction of destabilization of microtubules *in vitro*. These events lead to destruction of the microtubule networks beneath adherent bacteria on epithelial cells (Matsuzawa et al., 2004; Hardwidge et al., 2005). Furthermore, proteins extracted from EPEC-infected cells showed a corresponding loss of α-tubulin. Additionally, ectopic expression of EspG in epithelial cells caused microtubule disruption. Interestingly, mutation of both *espG* and *espG2* significantly delayed the kinetics of TEER drop and complementation of this double mutant with *espG* alone restored the EPEC-induced decrease in TEER (Tomson et al., 2005). The distribution of occludin from TJs was used as a marker of TJ integrity in Caco-2 cells infected with EPEC wild type and with a double mutant in *espG* and *espG2*. The double mutant induced a lesser amount of occludin accumulation in the cytoplasm than EPEC wild type. The impact of EspG on the TJs was not only restricted to occludin; ZO-1 and claudin-1/2 were also disturbed. Furthermore, to evaluate the role of EspG in the TJ restoration in calcium switch assays, Caco-2 monolayers were infected with *espG* double mutant and wild type strains. Δ*espG1/G2*-infected monolayer significantly recovered more barrier function (78 vs. 62% in wild type infected cells), suggesting that intact microtubule networks promote TJ recovery after EPEC infection (Glotfelty et al., 2014). These data demonstrate that occludin traffics on microtubules and that microtubule disruption perturbs TJs structure and function.

Golgi apparatus dispersion is a well-known indicator of the loss of microtubule network, and interestingly, EPEC EspG has been located into the Golgi apparatus (Selyunin et al., 2011; Glotfelty et al., 2014). Similar results were found using EHEC EspG. EHEC EspG interacts with GM130, a protein strongly attached to *cis*-Golgi membrane that is also related to maintaining the structure of the *cis*-Golgi (Clements et al., 2011). Additionally, it has been notice that Golgi destabilization prevents the traffic of membrane receptors in cell infected with EHEC (Clements et al., 2014). In the context of infection, epithelial cells infected with the mutant strain (Δ*espG/espG2*) retained giantin staining, a Golgi specific marker, predominantly perinuclear. In contrast, infection with the wild type strain induced the dispersion of Golgi marker (Glotfelty et al., 2014). Thus, EspG may use various alternatives for disrupting the TJ integrity; either by disruption of microtubule network, or by inhibiting the traffic of new TJ proteins from Golgi apparatus to plasma membrane. More studies are needed for further understand the mechanism of TJ disruption mediated by EspG and its relationship with the diarrhea produced by EPEC/EHEC.

## Non-LEE encoded effectors

Genes for the non-LEE encoded effectors are clustered in six pathogenicity islands along the genome. The genes of these effectors are found flanked by genes related to phages and/or transposons, implying that they were acquired by horizontal transfer (Dean and Kenny, 2009).

### NleA

The first identified non-LEE-encoded effector of A/E pathogens that is secreted by the T3SS was NleA (Non-LEE encoded effector A). It was firstly described in EHEC O157:H7 and then in all other A/E pathogens, including EPEC and *C. rodentium* (Gruenheid et al., 2004). NleA directly interacts with NLRP3 and affects the deubiquitination of NLRP3, a sensor molecule that has a central role in the inflammasome formation. This interaction reduces NLRP3 deubiquitination and the inflammasome formation. Accumulation of ubiquitinated NLRP3 decreases the activation of caspase-1 and the secretion of inflammatory IL-1β cytokines related with amplifying inflammation (Yen et al., 2015). During the inflammation process, disruption of TJ strands is one of the major contributions during the pathogenesis of intestinal diseases; mucosal immune activation is altered in response to increased epithelial permeability (Edelblum and Turner, 2009).

Although, NleA produces a reduction in the inflammatory response during EPEC infection, this effector also produces a contrary phenomenon that is TJ disruption. A NleA-dependent decrease in TEER and concurrent disruption of TJ strands was observed in EPEC-infected epithelial cells. In Caco-2 cells infected with EPEC wild type, ZO-1 and occludin redistribution was detected but not in cells infected with EPECΔ*nleA*, indicating that NleA is required for disruption of TJ strands during EPEC infection (Thanabalasuriar et al., 2010b). In order to cause TJ disruption, NleA primarily needs to direct to the Golgi apparatus. NleA has been located inside the Golgi and its localization in the Golgi depends on a consensus class I PDZ binding motif, ETRV, which is conserved among all A/E pathogens (Lee et al., 2008).

Once in the Golgi, NleA interacts and inhibits COPII (Coat complex protein) functions. COPII complex is formed by GTPase Sar1, the inner layer coat components Sec23 and Sec24, and the outer layer coat components Sec13 and Sec31. These subunits conjunctly work to produce vesicles in the exit sites of the endoplasmic reticulum, while specific cargo selection is mainly executed by Sec21 (Venditti et al., 2014). Recently, it has been found that a key component of the TJ integrity is the traffic mediated by COPII. Knock-down of COPII components in epithelial cells is sufficient to cause delocalization of ZO-1 and occludin from the epithelial cell periphery, indicating that the replacement of TJ proteins is essential to maintain its integrity (Thanabalasuriar et al., 2013). During EPEC infection, it has been found that NleA compromises the Sec23/Sec24 complex through the interaction with Sec24 subunit. While NleA expression in CHO cells reduces 50% the secretion of alkaline phosphatase, as a result of manipulation of the secretion of proteins in host cells, and NleA interacts with the four paralogs of Sec24 (Sec24A, Sec24B, Sec24C, and Sec24D) (Kim et al., 2007). The last 40 amino acids of the C-terminal of NleA are essential for these interactions (Venditti et al., 2014). Within this region, two motifs are important for binding the different Sec24 paralogs: (i) the amino acids 435–437 (residues IIQ) are crucial for Sec24A/B interaction, but less important for interaction to Sec24C/D; (ii) contrary, mutation of isoleucines 414 and 416 to alanine had a large effect on NleA-Sec24C/D interaction, but do not affect interaction with Sec24A/B. The NleA mutants NleAI414A/I416A and NleAI414A/I416A/ΔIIQ transfected in HeLa cells do not co-localized with the giantin Golgi marker as the native NleA does. Additionally, NleAI414A/I416A/ΔIIQ mutant showed a diminished binding to all four mammalian Sec24 paralogs (Thanabalasuriar et al., 2012).

Murine models have been useful tools to understand the role of NleA along A/E pathogen infection. Mice infected with a strain of *C. rodentium* engineered to express NleAI414A/I416A/ΔIIQ instead of native NleA was completely avirulent in mice, providing strong evidence that Sec24 interaction with NleA is critical for bacteria virulence (Thanabalasuriar et al., 2012). While the same susceptible C3H/HeOuJ mice infected with *C. rodentium* showed virtually no localized staining of claudin-3 in the TJs. Conversely, mice infected with Δ*nleA* or NleAI414A/I416A/ΔIIQ strains showed a claudin-3 staining localized in TJs, even at sites of bacterial adhesion. FITC-dextran permeability assays showed that mice infected with the wild type strain had significantly more FITC-dextran in their serum than those mice infected with Δ*nleA* or NleAI414A/I416A/ΔIIQ mutants. Similarly, mice infected with wild type strain had increased fecal water content and decreased amount of solid feces in their colons compared with uninfected mice. In contrast, mice infected with Δn*leA* or NleAI414A/I416A/ΔIIQ had solid fecal pellets in their colon and had similar fecal water contents as uninfected mice (Thanabalasuriar et al., 2013).

According to these data, NleA and its interaction with components of COPII complex promotes the disassembly of the TJ. And the disassembly is tightly correlated with the production of diarrhea in murine models.

### EspM

Tobe et al. exploited a bioinformatics approach to identify potential effectors encoded in the completed genome sequence of the EHEC Sakai strain, and found two additional members of the Map IpgB family (EspM1 and EspM2), both of which are translocated by the T3SS and encoded outside of LEE. EspM is highly frequent in EPEC and EHEC and belongs to effectors with WxxxE motifs, which have the capability for activating small GTases of Rho type (Arbeloa et al., 2009). Infection of Swiss 3T3 cells with EspM expressing EPEC induces stress fiber formation, and this depends on a conserved Trp residue (W70) of EspM, an effect that is shared between the two variants of EspM, EspM1 and EspM2. Additionally, the expression of a dominant negative of Rho inhibits the formation of stress fibers mediated by EspM. These results indicate that EspM modulates the actin dynamics through the activation of the Rho signaling pathway (Arbeloa et al., 2008). Arbeloa et al. demonstrated a direct binding of EspM to RhoA. On the other hand, the increase of free GTP or GDP inhibit the formation of EspM/RhoA complex in a concentration dependent way. The interaction between EspM and RhoA increases the nucleotide exchange, corroborating the GEF activity displayed by EspM.

During the infection, EspM acts as a repressor in the development of pedestals. Simovitch et al. found an increase in the size of pedestals induced by an EHEC mutant strain in *espM1* and *espM2* genes. On the other hand, an EPEC strain expressing *espM1* and *espM2* genes was unable to develop pedestals, involving to EspM in pedestal formation. Infection of polarized MDCK monolayers grown on coverslips with wild type EHEC resulted in marked alterations in the architecture of the cell monolayer, including the appearance of cells that bulge out of the monolayer and assume a round morphology. In contrast, the EHEC *espM* double mutant failed to do so. Additionally, monolayers infected with EspM2 expressing EPEC frequently assumed an abnormal round shape, with the main mass of their cell body bulging out. ZO-1was frequently found to be delocalized to more basal regions of the polarized cells (Simovitch et al., 2010). Additionally, ectopic expression of EspM in MDCK cells also showed this characteristic phenotype produced by EspM (Simovitch et al., 2010).

## Cooperative action of effectors during diarrhea induction by EPEC and EHEC

Effectors, either encoded inside or outside of LEE, manipulate the host cell signaling by manifesting a high level of multifunctionality, allowing EPEC and EHEC to colonize, multiply, and cause diarrhea. In previous years, the processes seen during infection with these bacteria were attributed only to independent action of one of its multiple effectors. Today, this concept has changed thanks to the discoveries of the multifunctional nature of these effectors, which has allowed the approach of new models of infection for EPEC and EHEC. These new models have been built based on redundant, synergistic, or antagonistic relationships of some effectors working in a coordinated spatial and temporal manner on different host organelles and cellular pathways during infection (Wong et al., 2011). The first report of a synergistic activity related to the destabilization of TJ strands, and consequently the production of diarrhea, strongly suggests that Map and EspF synergistically act for destabilizing the TJs. Since in the absence of one or another there is no redistribution of occludin and only a minor loss of TEER is induced in Caco-2 cell monolayers. Contrary, when both proteins are expressed in EPEC wild type strain, the effect of these effectors on the TJs (occludin redistribution) and on the TEER are increased even more that in strains where they are individually mutated these genes (Dean and Kenny, 2004). Although, these molecules appear to act synergistically, they retain some barrier disrupting activity in the absence of each other and thus displaying partial redundancy. However, in a murine model using *C. rodentium* as the pathogen agent and comparing with Map, EspF is more relevant for disassembly of TJs (redistribution of claudin-1, claudin-3, and claudin-5; Guttman et al., 2006).

In 2010, Thanabalasuriar et al. reported that NleA, EspF, and Map manifest a synergistic action on T84 cell monolayers. The TEER of monolayers infected with the EPECΔ*nleA* strain decreased 27%, as those infected with the EPECΔ*espF* strain. When the cell monolayers were infected with the EPECΔ*nleA* strain complemented with *nleA* gene, this strain recovered the capability to decrease the TEER in a 71%, a similar to the wild type strain infection. These data indicate that NleA is as important as EspF in the TJ disruption. The hypothesis of the synergism of these effectors was tested when the deficiencies in these effectors were complemented *in trans* for producing TJ disassembly. Cells co-infected with EPECΔ*nleA* and EPECΔ*espF* showed a decrease in the TEER similar to the cells co-infected with EPECΔ*nleA* and EPECΔ*map* strains. Similarly, the redistribution of the proteins ZO-1 and occludin was inhibited if one of these three proteins was lacking, and contrarily, this redistribution was recovered using the same combination of strains in co-infection (Thanabalasuriar et al., 2010b). From these data, a new model for the synergistic effect of EPEC/EHEC effectors related to TJ disassembly has been proposed. In this model, in uninfected cells some of the TJ proteins are delivered to TJ structures through the COPII-dependent pathways. When cells are infected with EPEC wild type, the EspF and Map activity result in the removal of proteins from TJs, while NleA blocks the delivery of newly synthesized TJ proteins by the inhibition of COPII. In cells infected with EPECΔ*espF*, NleA blocks the delivery of newly synthesized TJ proteins by inhibiting COPII, but TJs are not disassembled due to a lack of EspF/Map activity. In cells infected with EPECΔ*nleA*, EspF, and Map activity result in the removal of proteins from TJs but TJs are repaired via the delivery of newly synthesized TJ proteins using COPII-dependent pathways (Thanabalasuriar et al., 2010a). Although the mechanism used by these effectors to generate TJ disruption is poorly understood, it can be noticed that they exhibit an particular “all or nothing” phenotype, in which, if one of these three effectors is eliminated the ability of the bacteria to disrupt TJs is impaired. Therefore, the exact mechanism used by each individual effector may be very elusive (Thanabalasuriar et al., 2013).

## Remarkable conclusions

Mucosal surfaces are a portal of entry for many pathogens and the intestinal epithelium represents a surface of about 400 m^2^. In the case of extracellular enterobacteria one of the main challenges is to manipulate the selectively permeable barrier that represent the intestinal epithelium in order to colonize and survive. As extracellular bacteria the paracellular pathway might be a relevant target rather than the intracellular pathway. The paracellular pathway implies the cell junction, and the epithelial cells are held together by the apical junctional complexes, consisting of AJs and TJs, and by underlying desmosomes. Among these cell junctions, EPEC and EHEC mainly affect the TJs. Although the TJ structure is a large protein complex, the major types are the claudins and the occludins. These are associated with different peripheral membrane proteins, such as ZO-1 located on the intracellular side of plasma membrane, which anchor the strands to the actin component of the cytoskeleton. Thus, TJs join together the cytoskeletons of adjacent cells. One of TJ functions is helping to maintain the polarity of cells by preventing the lateral diffusion of integral membrane proteins between the apical and lateral/basal surfaces, allowing the specialized functions of each surface to be preserved. Thus, TJ integrity depends on various cell functions, such as actin cytoskeleton as mentioned above, microtubule network for vesicular trafficking including endocytosis of TJ proteins for replacement, membrane integrity, inflammation, and cell survival. EPEC and EHEC effectors target most of these functions. Interestingly, LEE and non-LEE effectors disrupt the TJ strands through indirect mechanisms of TJ disassembly by interfering with occludin dephosphorylation, actin rearrangement, microtubule network disruption, regulation of actin polymerization and disruption of vesicular trafficking (Figure 2). Nevertheless, EPEC and EHEC exploit the TJ dynamics to open them, and in consequence, the bacteria cause diarrhea. EPEC and EHEC secrete effectors classified as multitask proteins, indicating that they are not only related to the production of diarrhea but also with the manipulation of other cellular processes. Remarkably, these bacterial effectors mimic host proteins to manipulate the signaling pathways, for instance acting as GEF, Rho, etc. Thus, new regions or motifs inside these effector proteins, which are related to the manipulation of cellular processes, are constantly described revealing new ways for which EPEC and EHEC exploit cellular processes for bacterial colonization, adhesion, and propagation.

In the past decade, it has been clear that the integration of the effects of multiple effectors (LEE and non-LEE effectors) is a difficult task. Most of the effectors protein contains functional motifs, which mimicking cell functions, such as binding motifs to SH3 or PDZ domains. Most effectors are multifunctional proteins and EspF is one of the best examples, which can act in the cytoplasm, in the mitochondria or in the nucleolus. Furthermore, the multifunctional nature of these effectors have allowed to build new models based on redundant, synergistic or antagonistic relationship of some effectors working in a coordinated spatial and temporal manner on different host organelles and cellular pathways during EPEC and EHEC infection.

## Author contributions

PU-S, OG-L, FN-G contributed to the writing of this review. All authors read and approved the final manuscript.

### Conflict of interest statement

The authors declare that the research was conducted in the absence of any commercial or financial relationships that could be construed as a potential conflict of interest.
